# Reversible splenial lesion syndrome type II in youth mimicking acute ischemic stroke like onset: A case report

**DOI:** 10.1097/MD.0000000000034568

**Published:** 2023-08-04

**Authors:** Yan Li, Zhengyang Wang, Sijia Lai, Manfei Li, Huihui Liang, Hui Qin, Kaihua Wang

**Affiliations:** a Guangxi International Zhuang Medicine Hospital, Nanning, China; b Guangxi University of Traditional Chinese Medicine, Nanning, China.

**Keywords:** acute ischemic stroke, corpus callosum, reversible splenial lesion syndrome

## Abstract

Reversible splenial lesion syndrome (RESLES) is a newly recognized syndrome. Its typical pathologic findings is a reversible progress correlated with transiently reduced diffusion lesion in the splenium of the corpus callosum. The common clinical symptoms include mildly altered states consciousness, delirium, and seizure.

**Methods:**

We presented a 21-year-old patient with signs of acute ischemic stroke (AIS), including symptoms of weakness on the right upper limb and aphasia, lasting 50 minutes until he was taken to the emergency. He just had a cough 20 days ago.

**Results:**

An elevated level of white blood cell count, neutrophil count, monocyte count, protein of cerebrospinal fluid was found in laboratory examinations. Magnetic resonance imaging revealed distinct lesions involving white matter in the splenium of the corpus callosum and frontal-parietal cortex on both cerebral hemispheres. Digital subtraction angiography examination was also unremarkable. The patient recovered to baseline within 4 days. We treated the patient with glucocorticoid, antiviral drugs, butylphthalide, and dehydrating drugs. In addition, the follow-up brain magnetic resonance imaging scan showed reduced lesions. AIS-like symptoms did not occur during a 30-day follow-up period.

**Conclusion:**

This patient with reversible splenial lesion syndrome type II exhibited AIS-like symptoms, which was uncommon on clinical. This case extends the recognized clinical phenotypes for this disorder.

## 1. Introduction

Reversible splenial lesion syndrome (RESLES) is a newly minted syndrome that was first described by Tada et al in 200 RESLES was first reported by Garcia-Monco et al in 2011.^[1]^ It is a new clinicoradiological syndrome characterized by transiently reduced diffusion lesion in the splenium of the corpus callosum (SCC).^[2]^ The typical clinical symptoms of RESLES include mildly altered states consciousness, delirium, and seizure. The most common etiology and essential pathogenesis of RESLES were viral infection and reversible cytotoxic edema, respectively.^[3]^ The common feature of the syndrome is the disappearance of imagine abnormities and improvement of clinical in a few days. It was further proposed to be classified into RESLES type I, with an isolated lesion in the SCC, and RESLES type II, with an combination of reversible lesion in the SCC and in other brain areas. We report here a young patient with RESLES type II, manifesting the acute ischemic stroke (AIS) like symptoms.

## 2. Case report

A 21-year-old young man was admitted to our hospital with a symptom of weakness on the right upper limb and aphasia in 50 minutes. The pervious history is unremarkable except a cough 20 days ago, without fever. The cough symptom was resolved with a week intravenous fluid therapy, but the specific medication was unknown.

An elevated level of white blood cell count (10.34 × 10^9^/L, normal range: 3.5–10 × 10^9^/L), neutrophil count(6.88 × 10^9^/L, normal range: 1.8–6.3 × 10^9^/L), monocyte count (0.71 × 10^9^/L, normal range:0.1–0.6 × 10^9^/L) was found in complete blood cell count. No evidence of abnormity was found in other laboratory examinations, including blood coagulation, electrolytes, renal function, liver function, myocardial enzyme spectrum. We performed cranial CT on the young man immediately and found no high density lesion, indicated that cerebral hemorrhage could be excluded. We preliminary considered the patient to be AIS. The patient’s NIHSS score was 12 and without any contraindications to thrombolytic therapy. We executed thrombolytic therapy to the patient after his family signed the informed consent at 2 hours after the onset of the symptom. The weakness on the right upper limb and aphasia of the patient recovered completely after rt-PA consumed. Then, we performed digital subtraction cerebral angiography to the patient. Digital subtraction angiography showed no intracranial and extracranial large arteries’ stenosis or occlusion. Brain magnetic resonance imaging (MRI) was conducted to the patient after digital subtraction angiography.

**Figure 1.**
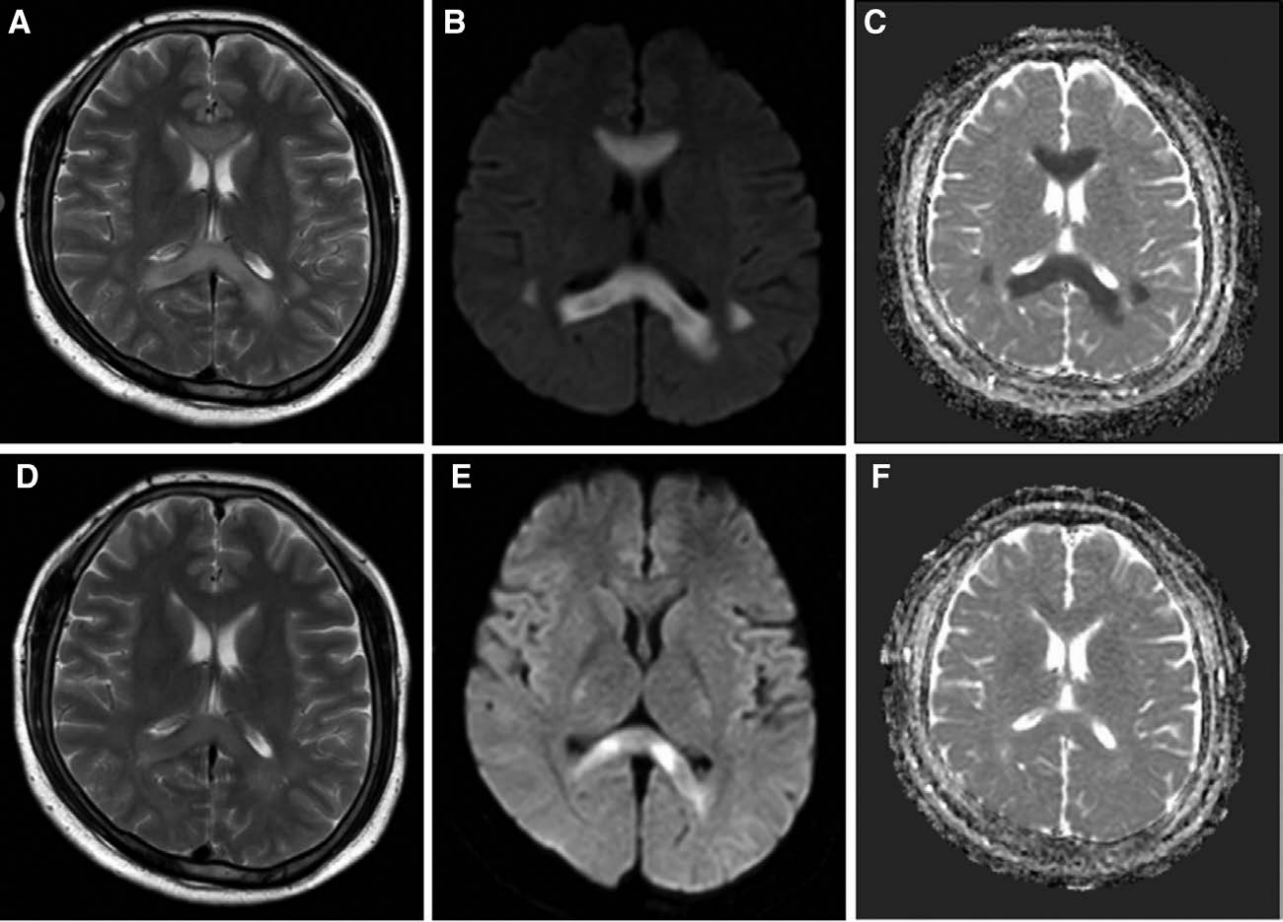
MRI imaging at 6 hours following the onset of symptoms showing increased signals on T2-images in the SCC and frontal-parietal subcortex of both cerebral hemispheres (A). Abnormal signals were also found at DWI (B) and ADC images (C) in the same area. MRI imaging at 8 days later exhibited reduced abnormal signals in T2 (D), DWI (E), and ADC (F) in the same area. ADC = apparent diffusion coefficient, DWI = diffusion-weighted imaging, MRI = magnetic resonance imaging, SCC = splenium of the corpus callosum.

**Figure 2.**
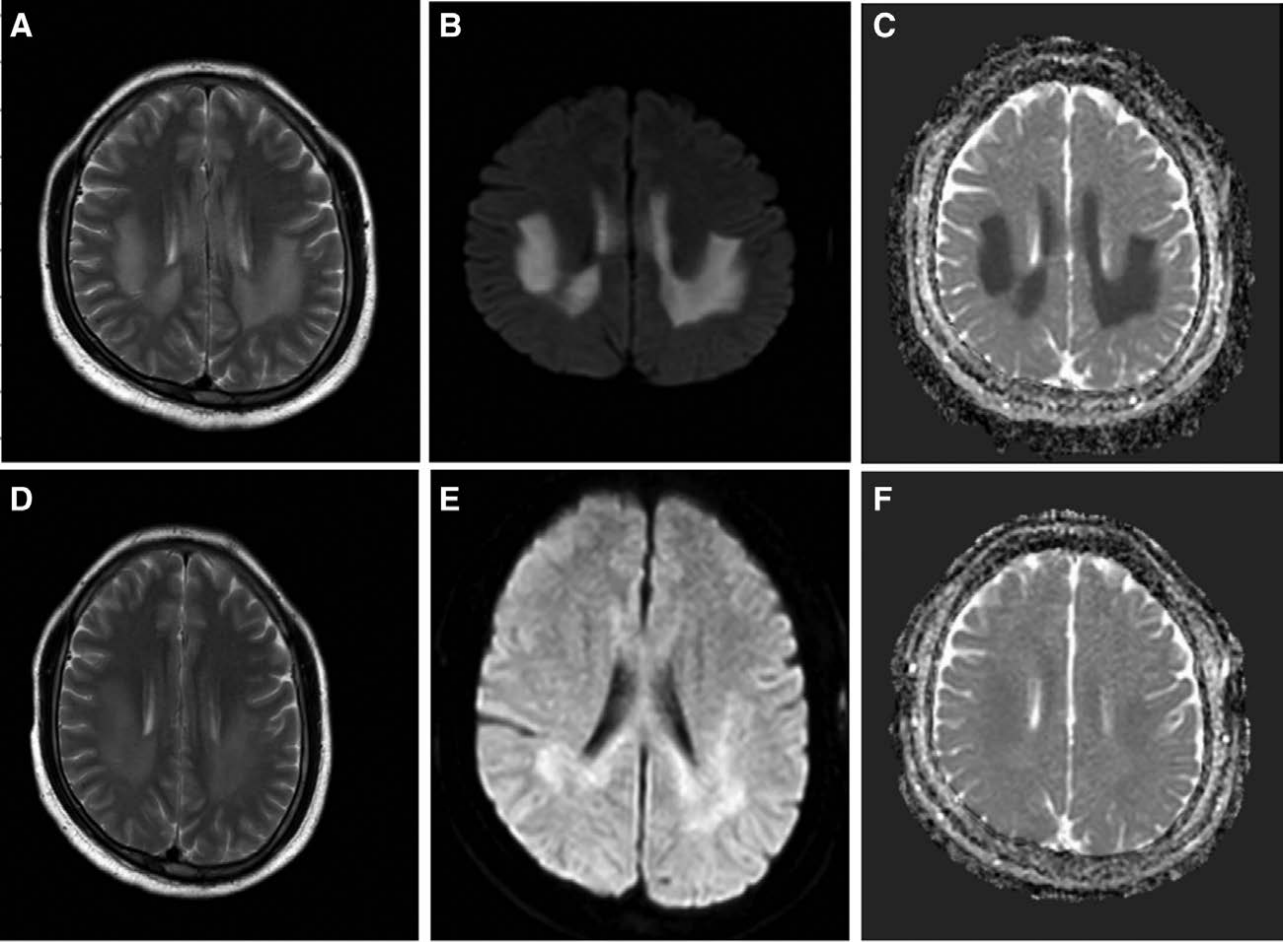
MRI imaging at 6 hours following the onset of symptoms showing increased signals on T2-images in the SCC and frontal-parietal subcortex of both cerebral hemispheres (A). Abnormal signals were also found at DWI (B) and ADC images (C) in the same area. MRI imaging at 8 days later exhibited reduced abnormal signals in T2 (D), DWI (E), and ADC (F) in the same area. ADC = apparent diffusion coefficient, DWI = diffusion-weighted imaging, MRI = magnetic resonance imaging, SCC = splenium of the corpus callosum.

The first MRI images (T2) revealed distinct lesions involving white matter in the SCC and frontal-parietal cortex on bilateral cerebral hemispheres approximately 6 hours following the onset of symptoms (Fig. 1). These lesions showed restricted diffusion with hyperintense signal on both diffusion-weighted imaging (DWI) and apparent diffusion coefficient (ADC) sequences. There was no contrast enhancement. A follow-up brain MRI was performed 8 days after symptoms onset showed reduced size of lesions with decreased signals in T2, DWI, and ADC (Fig. 2).

Cerebrospinal fluid examination was conducted to test viral encephalitis and autoimmune encephalitis. The results could rule out the 2 diseases. The white blood cell count (5 × 10^6^/L), glucose (3.47 mmol/L) and chloride (125 mmol/L) were within normal ranges. The protein (0.5 g/L, normal range:0.2–0.45 g/L) was slightly beyond the normal range. The electroencephalogram examination was normal. An enzyme immunoassay on serum showed that rubella virus IgG antibody, cytomegalovirus IgG antibody were positive and other virus markers with herpes simplex virus, influenza virus and adenovirus were negative. Cervical and thoracic vertebrae MRI found no abnormal signals. Abdominal, cardiac ultrasound, and chest X-ray were normal.

**Figure 3.**
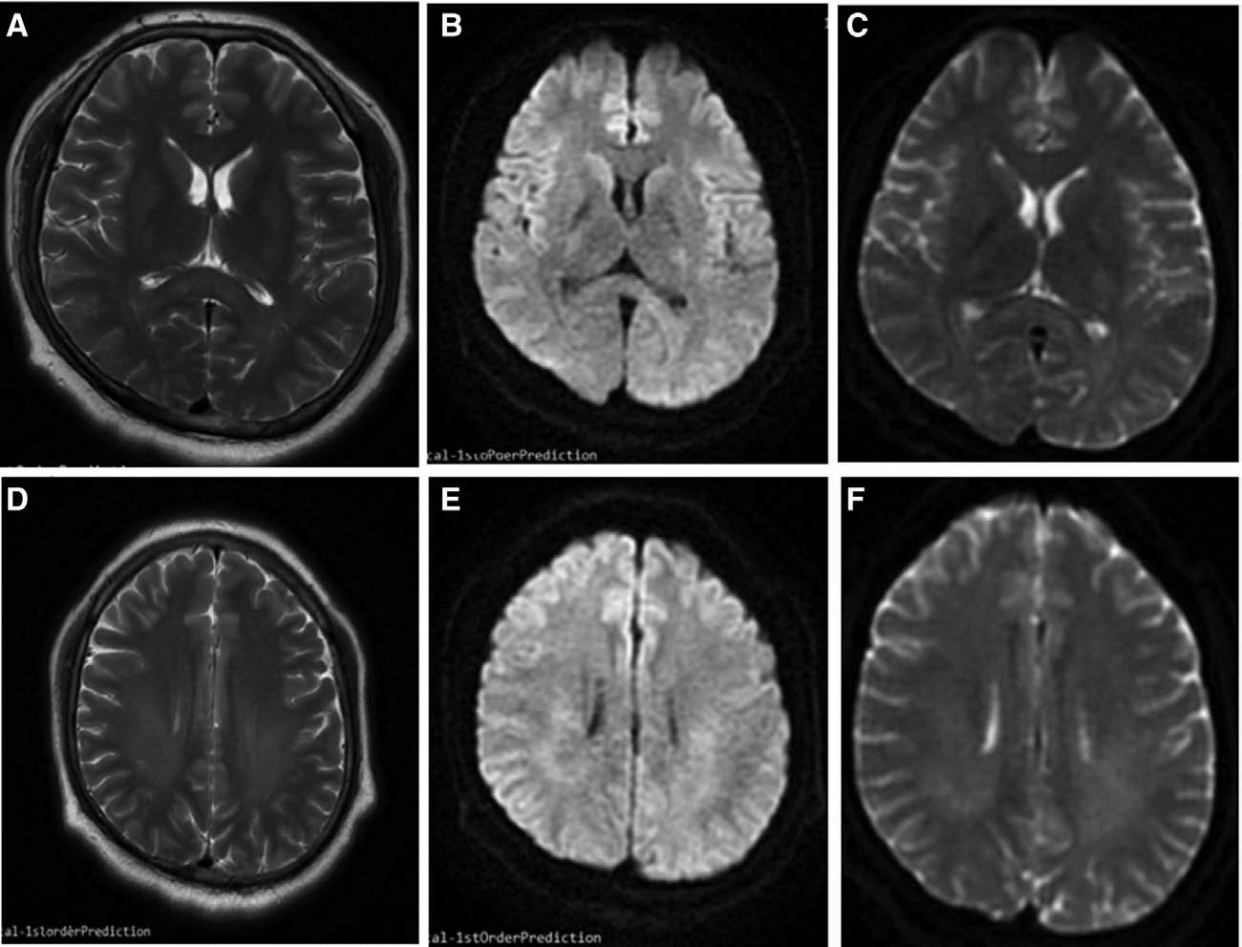
A follow-up brain MRI was performed 30 days discharged from hospital, and the lesions determined on the second MRI were significantly regressed in T2, DWI and ADC (A, B and C, D, E and F). ADC = apparent diffusion coefficient, DWI = diffusion-weighted imaging, MRI = magnetic resonance imaging.

The symptoms of weakness on the right upper limb and aphasia recurrent twice within 3 days after hospital admission. Symptoms lasting about 1 hour each time mimicking transient ischemic attack. We treated with glucocorticoid, antiviral drugs, butylphthalide, dehydrating drugs. The patient recovered completely at the fourth day and never recurrent until discharge from hospital. We followed up the patient after discharge from hospital 30 days later and conducted the third brain MRI to him. Symptom of weakness on the right upper limb and aphasia did not occur on the patient after discharge. The images of brain MRI revealed that the lesions determined on the second MRI were significantly regressed in T2, DWI, and ADC (Fig. 3).

## 3. Discussion

Tada et al reported a series of 15 patients with mild encephalitis/encephalopathy with reversible splenial lesion (MERS) in 2004.^[4]^ In 2011, Garcia-Monco et al^[1]^ searched the MEDLINE database from 1966 to 2007 and termed the presence of transient lesions involving the SCC RESLES.

Therefore, RESLES associated with encephalitis/encephalopathy was interchangeably termed MERS, sometimes.^[5]^ The pathogenesis of RESLES has not been clearly elucidated. Currently admitted pathogenesis including inflammatory infiltration, intramyelinic edema, immune system activation, axonal damage, and oxidative stress.^[6]^ All these pathological processes eventually led to cytotoxic edema.

Reversible splenial lesion and transient encephalopathy is indispensable hallmark of the diagnosis of the RESLES. In this case, we first showed AIS-like symptoms in a young man with RESLES type II. RESLES is classified as RESLES type I or RESLES type II, depending on the sites of lesions.^[7,8]^ Lesions in type I are seen only in the corpus callosum. Lesions in type II involve not only the splenium of corpus callosum, but also the splenium of knee and body, even involve the deep or subcortical white matter. RESLES type II is a rare occurrence compared to type I. RESLES type II patients generally were children and young people, and they usually had a good prognosis based on previous reports.^[9]^

Clinically, variable symptoms have been reported for RESLES, including disorders of consciousness, dysphonia, seizure, or delirium.^[10]^ Symptoms of RESLES also included fever, ataxia, headache, vomiting, or diarrhea. Little has been reported in the literature about RESLES only presenting as AIS-like episodes. Dong et al^[6]^ reported a 14-year-old girl who presented as triple episodic weakness on the right upper limb, slurred speech, and bucking, lasting several hours in each time in 2016. The girl had a slight cold 2 weeks ago, with loss of appetite, tiredness, and headache, without fever. Brain MRI demonstrated reduced lesions with decreased signals in corpus callosum and peripheral white matter after 10 days without any special treatment.

It is presumed that viral infection might be causative for the disease. Infection, especially viral infections is considered to be predisposing factor for RESLES. ^[3]^ Influenza viruses A and B are the most common causative viruses.^[11]^ In this report the patient presented with a previous history of cough which may supportive to the fact that he might have viral infection within the past 1 week.

The characteristics of literature report and this patient indicated that young patients with acute stroke-like attacks should be considered the possibility of RESLES. There is no specific treatment options exist for RESLES. We treated the patient with glucocorticoid, antiviral drugs, and dehydrating drugs.

In conclusion, this case demonstrates that RESLES type II can present acute stroke-like symptoms, which is not reported in literature before. Clinicians should be mindful with acute stroke-like symptoms in patients with possible RESLES type II.

## Acknowledgments

We thank the patient and his family for their consents to publish the case report and the accompanying his images.

## Author contributions

**Data curation:** Zhengyang Wang.

**Formal analysis:** Sijia Lai.

**Investigation:** Manfei Li.

**Methodology:** Huihui Liang.

**Project administration:** Hui Qin.

**Writing – original draft:** Yan Li.

**Writing – review & editing:** Kaihua Wang.

## References

[1] Garcia-Monco JC Cortina IE Ferreira E. Reversible splenial lesion syndrome (RESLES): what’s in a name? J Neuroimaging. 2011;21:e1–14. 18681931 10.1111/j.1552-6569.2008.00279.x

[2] Fu ML Han N Wang W. Cytomegalovirus-associated mild encephalopathy/encephalitis with reversible splenial lesion. Neurologist. 2021;26:172–4. 34491933 10.1097/NRL.0000000000000334

[3] Choi PK Yoon EJ Ha SW. Reversible splenial lesion syndrome caused by rubella infection. Neurol Asia. 2017;22:271–4.

[4] Tada H Takanashi J Barkovich AJ. Clinically mild encephalitis/encephalopathy with a reversible splenial lesion. Neurology. 2004;63:1854–8. 15557501 10.1212/01.wnl.0000144274.12174.cb

[5] Ka A Britton P Troedson C. Mild encephalopathy with reversible splenial lesion: an important differential of encephalitis. Eur J Paediatr Neurol. 2015;19:377–82. 25707871 10.1016/j.ejpn.2015.01.011

[6] Dong K Zhang Q Ding JP. Mild encephalopathy with a reversible splenial lesion mimicking transient ischemic attack: a case report. Medicine (Baltimore). 2016;95:e5258. 27858890 10.1097/MD.0000000000005258 PMC5591138

[7] Hudkins M O’Neill J Tobias MC. Cigarette smoking and white matter microstructure. Psychopharmacology (Berl). 2012;221:285–95. 22215225 10.1007/s00213-011-2621-9 PMC4111107

[8] Yuan J Yang S Wang S. Mild encephalitis/encephalopathy with reversible splenial lesion (MERS) in adults: a case report and literature review. BMC Neurol. 2017;17:103. 28545419 10.1186/s12883-017-0875-5 PMC5445341

[9] Gawlitza M Hoffmann KT Lobsien D. Mild encephalitis/encephalopathy with reversible splenial and cerebellar lesions (MERS type II) in a patient with hemolytic uremic syndrome (HUS). J Neuroimaging. 2015;25:145–6. 25729817 10.1111/jon.12089

[10] Hara M Mizuochi T Kawano G. A case of clinically mild encephalitis with a reversible splenial lesion (MERS) after mumps vaccination. Brain Dev. 2011;33:842–4. 21273018 10.1016/j.braindev.2010.12.013

[11] Iwata A Matsubara K Nigami H. Reversible splenial lesion associated with novel influenza A (H1N1) viral infection. Pediatr Neurol. 2010;42:447–50. 20472201 10.1016/j.pediatrneurol.2010.01.017

